# Influence of Protein Abundance on High-Throughput Protein-Protein Interaction Detection

**DOI:** 10.1371/journal.pone.0005815

**Published:** 2009-06-05

**Authors:** Joseph Ivanic, Xueping Yu, Anders Wallqvist, Jaques Reifman

**Affiliations:** Biotechnology HPC Software Applications Institute, Telemedicine and Advanced Technology Research Center, US Army Medical Research and Materiel Command, Ft. Detrick, Maryland, United States of America; Harvard University, United States of America

## Abstract

Experimental protein-protein interaction (PPI) networks are increasingly being exploited in diverse ways for biological discovery. Accordingly, it is vital to discern their underlying natures by identifying and classifying the various types of deterministic (specific) and probabilistic (nonspecific) interactions detected. To this end, we have analyzed PPI networks determined using a range of high-throughput experimental techniques with the aim of systematically quantifying any biases that arise from the varying cellular abundances of the proteins. We confirm that PPI networks determined using affinity purification methods for yeast and *Eschericia coli* incorporate a correlation between protein degree, or number of interactions, and cellular abundance. The observed correlations are small but statistically significant and occur in both unprocessed (raw) and processed (high-confidence) data sets. In contrast, the yeast two-hybrid system yields networks that contain no such relationship. While previously commented based on mRNA abundance, our more extensive analysis based on protein abundance confirms a systematic difference between PPI networks determined from the two technologies. We additionally demonstrate that the centrality-lethality rule, which implies that higher-degree proteins are more likely to be essential, may be misleading, as protein abundance measurements identify essential proteins to be more prevalent than nonessential proteins. In fact, we generally find that when there is a degree/abundance correlation, the degree distributions of nonessential and essential proteins are also disparate. Conversely, when there is no degree/abundance correlation, the degree distributions of nonessential and essential proteins are not different. However, we show that essentiality manifests itself as a biological property in all of the yeast PPI networks investigated here via enrichments of interactions between essential proteins. These findings provide valuable insights into the underlying natures of the various high-throughput technologies utilized to detect PPIs and should lead to more effective strategies for the inference and analysis of high-quality PPI data sets.

## Introduction

The accurate modeling of cellular processes requires knowledge of the underlying components together with practical descriptions of the interactions between them [1]. Proteins make up much of the cellular machinery; however, they may act individually, as parts of a dynamic pathway, or as elements of multi-component complexes that behave as individual functional entities [2]. The elucidation of protein roles is enhanced by discovery of their interactions with other proteins in the cell. Recent advances in experimental high-throughput (HT) technologies, most notably in the forms of the yeast two-hybrid (Y2H) [3] and tandem-affinity-purification (TAP) [4] platforms, have enabled large-scale protein-protein interaction (PPI) screens and subsequent constructions of corresponding PPI networks. A number of HT data sets, from these and other experimental platforms, are available for the yeast *Saccharomyces cerevisiae* [5]–[9] and for a small number of other species, including *Escherichia coli* [10], [11], *Drosophila melanogaster* [12], [13], and *Caenorhabditis elegans* [14].

Due to their potential significance in delineating biological organization, the topologies of PPI networks have been explored using a variety of graph-theoretical techniques [15]–[17]; however, recent investigations have found many of them to resemble probabilistic, or random, frameworks [18], [19]. PPI networks are also routinely exploited for the discovery of biological traits, where correlations among topological properties and biological attributes are probed for. Examples of inferred relationships include those between degree (number of interactions) and essentiality [20]–[23], and connectivity and evolutionary rate [24]–[26]. Other studies aim to identify biological entities, such as functional modules [27]–[29] and pathways [30], [31], in the networks. More recently, PPI network information has been used to augment gene expression measurements to identify condition-specific response complexes [32], [33]. PPI networks also have prospective roles in drug discovery [34].

It is clear that PPI networks have the potential to considerably supplement many areas of biological research. However, it is well known that data sets from different studies have very small numbers of coincident interactions [6], [35]. These small overlaps have led to some skepticism and suggestions of bias regarding their authenticities [36], [37]. The aforementioned observations and reservations were primarily based on analyses of three experimental studies of yeast proteins, two using Y2H screens [7], [9] and another using a HT mass spectrometric protein complex identification (HMS-PCI) technique [6], which is based on an affinity purification procedure. More recently, two large independent yeast PPI data sets determined using nearly identical TAP methodologies have become available [5], [8]. While the number of mutually detected TAP interactions is modest, the overlaps of the TAP-observed interactions with the Y2H and HMS-PCI data sets are very small. A very small interaction overlap also exists between two TAP data sets of *E. coli* [10], [11].

For PPI networks to be effectively utilized, their authenticities must be established. Platform-dependent high-quality interaction maps for yeast have recently been deduced for TAP [38] and Y2H [39] methodologies. However, a major step toward extracting and verifying credible interactions from raw experimental data requires comprehension of the distinct systematic biases present in the various experimental platforms. Previous investigations for yeast have suggested that protein abundance is an important factor for detecting interactions in affinity purification studies but not in Y2H screens [35], [40]. Von Mering et al. [35] showed that in PPI data sets deduced from two affinity purification studies (TAP [41] and HMS-PCI [6]), proteins having more interactions were more likely to have larger corresponding messenger RNA (mRNA) abundances while no such bias was detected in a PPI data set deduced from Y2H screens. Björklund et al. [40] showed that PPIs detected by two more-recent TAP studies [5], [8] were enriched with highly-abundant (>6000 molecules/cell) proteins, while a Y2H data set contained no significant enrichment. Although each study confirmed an abundance effect in affinity purification experiments for yeast, they did not perform comprehensive studies investigating the total extent of any abundance influence. Simply considering the impact of only highly-abundant proteins is insufficient to ascertain the scope of any abundance effects. Most proteins in a cell do not have very high abundances; therefore, it is useful to probe whether relative levels of promiscuity, possibly stemming from the varying abundances of the proteins, are perceivable in a variety of affinity purification data sets, including those that are inferred high-quality.

The influence of protein abundance upon the method of interaction detection is reinvestigated here. We analyzed PPI data sets encompassing three different platforms by incorporating cellular protein as well as mRNA abundance levels measured using three diverse technologies: western blot (WB) [42], flow cytometry (FC) [43], and gene expression (GE) [44]. Together with yeast PPI data sets examined in a previous study [35], we also investigated more recent TAP data sets for yeast [5], [8] and *E. coli* [10], [11]. Correlations between protein degree, or number of detected interactions, and cellular protein and mRNA abundances were determined with no averaging or binning of data. Additionally, to gauge the potential for artificial correlations arising from irregular abundance distributions we computed distributions for proteins by degree. We find that all TAP and HMS-PCI PPI data sets for yeast and *E. coli* contain a statistically significant correlation between protein degree and cellular abundance, while the Y2H data sets show no such relationship. The findings confirm that affinity purification methods are influenced by probabilistic interactions due to differences in protein concentrations. While it is known that the nature of affinity purification methods induce retrieval of nonspecific contaminants, or promiscuous prey proteins, we find that their promiscuity is related to their high abundance. While these results may not be unexpected, here we quantify the levels of the abundance effects and show their persistence throughout the data sets. More interestingly, analysis of high-confidence (HC) interaction data sets inferred in the affinity purification studies show that they, too, have a statistically significant correlation between degree and abundance. As mentioned earlier, the Y2H data sets, including HC, show no correlation between degree and abundance. Therefore, we substantiate here a systematic difference between PPI networks determined from Y2H and affinity purification methods.

In light of the discovered associations between degree and abundance, we reinvestigated the centrality-lethality rule [20]–[23], which implies that higher-degree proteins are more likely to be essential. We find, through strict statistical analyses of degree distributions of essential and nonessential proteins, that the raw and HC Y2H data sets show no correlation between degree and essentiality, while the HMS-PCI and TAP PPI networks, with one exception, contain substantial correlations. However, it is also found that essential proteins are generally more abundant than nonessential proteins and, therefore, these latter correlations may be artificially induced. In fact, we generally find that degree/abundance and degree/essentiality correlations occur in tandem where either both are present or both are absent. As such, the centrality-lethality rule may be misleading. In an effort to identify nonrandom signatures in the interaction data sets we determined, via comparisons with strict randomized simulations, the propensity for essential proteins to selectively interact with each other. We find that all yeast PPI datasets contain significant enrichments of essential-essential interactions. While the propensity for essential proteins to be involved in essential complex biological modules has been realized previously in HC networks [23], we demonstrate the more general case that essential proteins prefer to interact with each other.

These findings provide valuable insights into the underlying natures of and the differences between the various HT technologies utilized to detect PPIs. This knowledge should lead to more effective strategies for the inference and analysis of high-quality PPI data sets.

## Materials and Methods

We analyzed protein interaction networks for yeast and *E*. *coli* determined from Y2H [7], [9], HMS-PCI [6], and TAP [5], [8], [10], [11] platforms. These studies provide lists of all experimentally observed interactions. These *unprocessed* data sets are referred to here as *raw*. In all cases, raw binary interactions (non-self and undirected) and subsequent PPI networks were assembled by tabulation of bait-prey pairs. Some of these studies additionally attempt to identify substantive interactions using a range of methodologies, including experimental reproducibility, removal of suspect promiscuous proteins, and assignment of confidence scores using computational techniques. These latter data sets are referred to here as *high confidence*. We have investigated raw PPI networks and any corresponding HC data sets that were concurrently inferred.

### Yeast Data

Raw Y2H data sets from two investigations, labeled *Ito* [7] and *Uetz* [9], were downloaded from the IntAct database [45] (http://www.ebi.ac.uk/intact/site/index.jsf). Ito et al. [7] additionally provide a core, or HC, data set which contains interactions that were experimentally detected at least three times and this was downloaded from (http://itolab.cb.k.u-tokyo.ac.jp/Y2H).

Purification data from one HMS-PCI study, labeled *Ho* [6], was acquired from the original publication. Ho et al. [6] also infer a HC data set by removal of suspect promiscuous prey proteins and this was downloaded from IntAct.

Purification data from two TAP investigations [5], [8] were acquired from their original publications. The raw data set of Gavin et al. [5], labeled *Gavin*, used matrix-assisted laser desorption/ionization-time-of-flight mass spectroscopy (MALDI-TOF MS) to identify co-purifying proteins. A corresponding HC data set was inferred in their study by first determining ‘socio-affinity’ scores for each pair of proteins followed by an iterative clustering procedure that was refined using a curated set of protein complexes [46]. Two raw data sets were taken from the study of Krogan et al. [8]. The first, labeled *Krogan-TOF*, used MALDI-TOF MS to identify co-purifying proteins, while the second, labeled *Krogan-LCMS*, used liquid chromatography tandem mass spectrometry (LCMS) for protein identifications. Two types of HC data sets from the study by Krogan et al. [8] were downloaded from (http://tap.med.utoronto.ca/downloads.php). Both of their HC data sets were inferred by first removing 44 nonspecific contaminants and nearly all cytoplasmic ribosomal subunits from the raw data. The first HC data set, labeled *Krogan-INT*, contains the remaining interactions that were identified by both detection methods. The second, labeled *Krogan-CORE*, was derived using machine-learning algorithms trained on curated protein complexes in the MIPS reference database [47].

Yeast cellular protein and mRNA abundances during normal aerobic growth were taken from three investigations, where each used a different measurement methodology: WB [42], FC [43], and GE analysis [44]. Essential yeast proteins were obtained from the Saccharomyces Genome Deletion Project (http://www-sequence.stanford.edu/group/yeast_deletion_project/Essential_ORFs.txt) and the Munich Information Center for Protein Sequences (MIPS) (ftp://ftpmips.gsf.de/yeast/catalogues/gene_disruption). Only proteins annotated as essential in both datasets were considered to be essential here.

### *Escherichia coli* Data

Raw TAP-determined PPI networks from two investigations, labeled *Butland* [10] and *Arifuzzaman* [11], were acquired from their original publications. Gene expression measurements during normal aerobic growth were taken from three studies [48]–[50].

### Computational Analyses

Correlations between protein degree and abundance were evaluated by determining Pearson and Spearman rank correlation coefficients for log(degree) vs. log_2_(abundance). In every case the two coefficients were very similar, with Spearman's correlation coefficient generally being slightly smaller in magnitude. Correlation analyses were performed for pairs of individual data sets (PPI network vs. abundance measurement set) with no averaging of data, i.e., a protein was included as a separate data entity if both its degree and abundance were known. To illustrate the general trends in the correlations, or lack of, we generated plots of log(degree) vs. <log_2_(abundance)>, where the latter quantity was determined by averaging log_2_(abundance) values, for a particular abundance measurement set, of proteins having the same degree.

**Figure 1:**
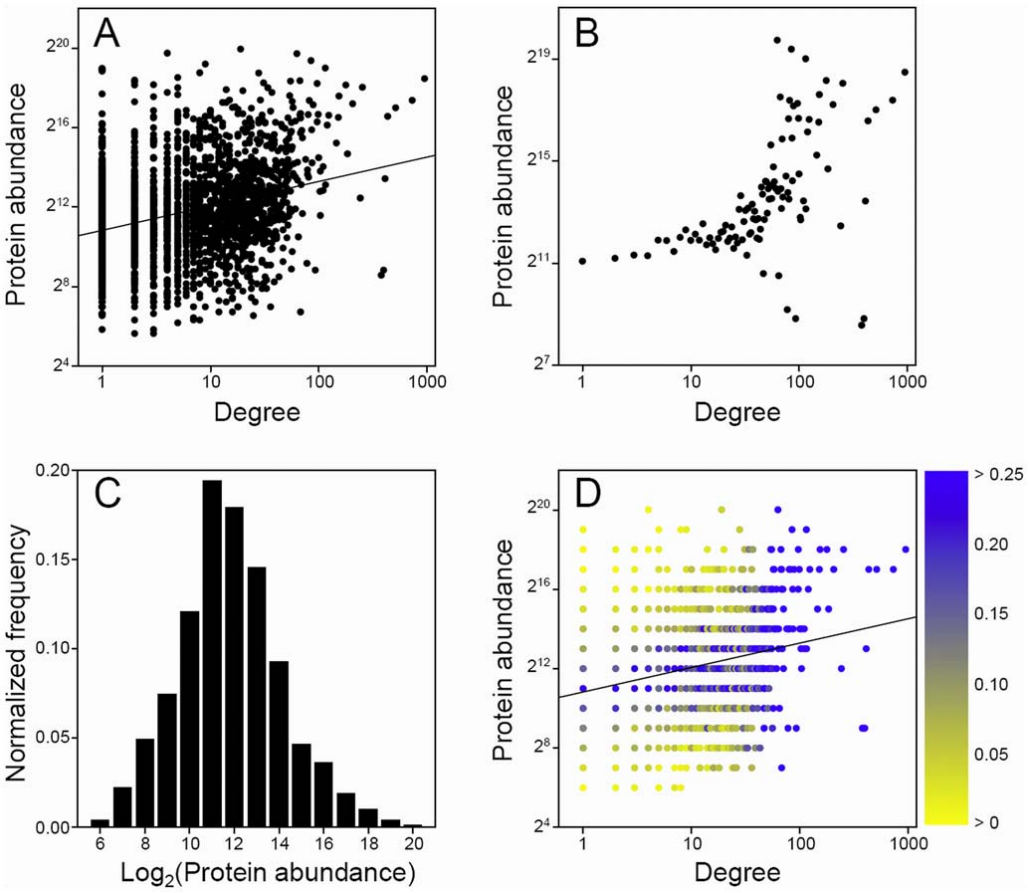
Relationship between protein degree and abundance in the raw yeast TAP Gavin PPI network [5] using the western blot abundance measurements of Ghaemmaghami et al. [42]. (A) All data points, i.e., each protein's degree and abundance is plotted; (B) averaged data where log_2_(abundance) values were averaged over proteins having the same degree; (C) total normalized abundance distribution, binned in integer values of log_2_(abundance), for proteins appearing in both PPI and abundance measurement data sets; (D) normalized abundance distributions for each degree where frequencies are shown by color: most yellow signifies smallest nonzero value and most blue represents values larger than 0.25. Best-fit line to data in (A) also shown in (D).

**Figure 2:**
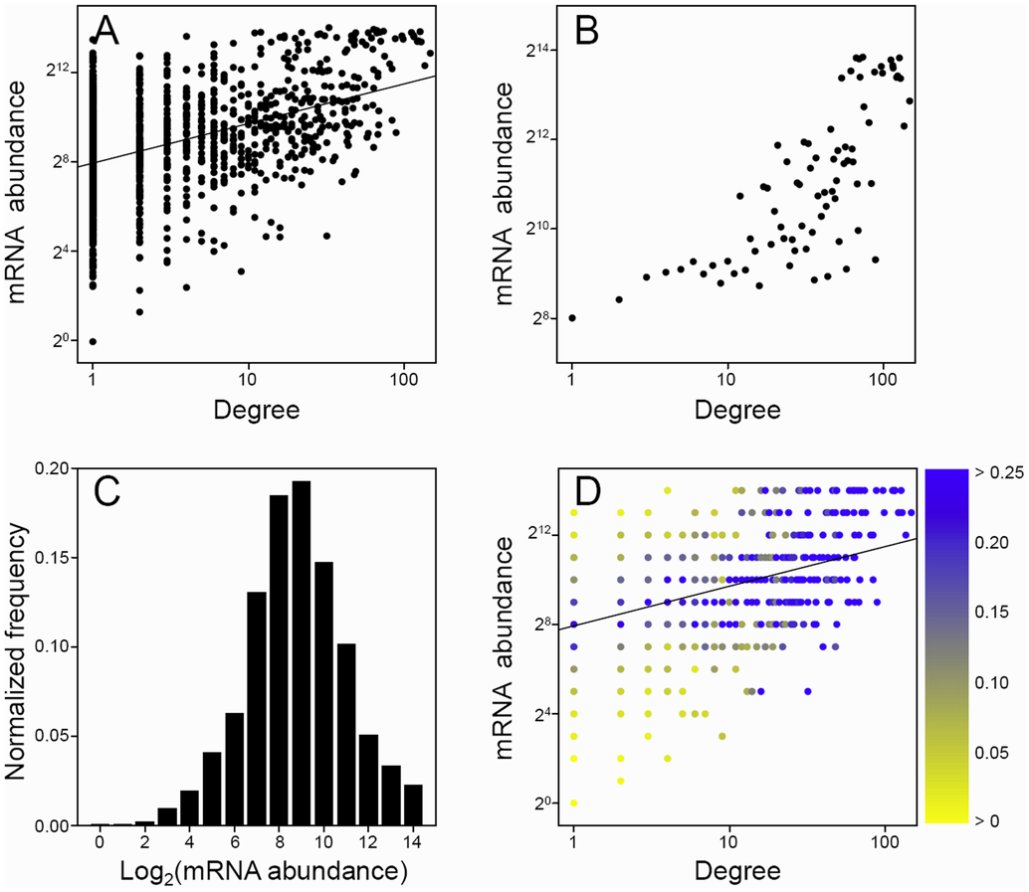
Relationship between protein degree and abundance in the raw *E. coli* TAP Butland PPI network [10] using the gene expression measurements of Covert et al. [48]. (A)–(D), see Figure 1 legend.

**Figure 3:**
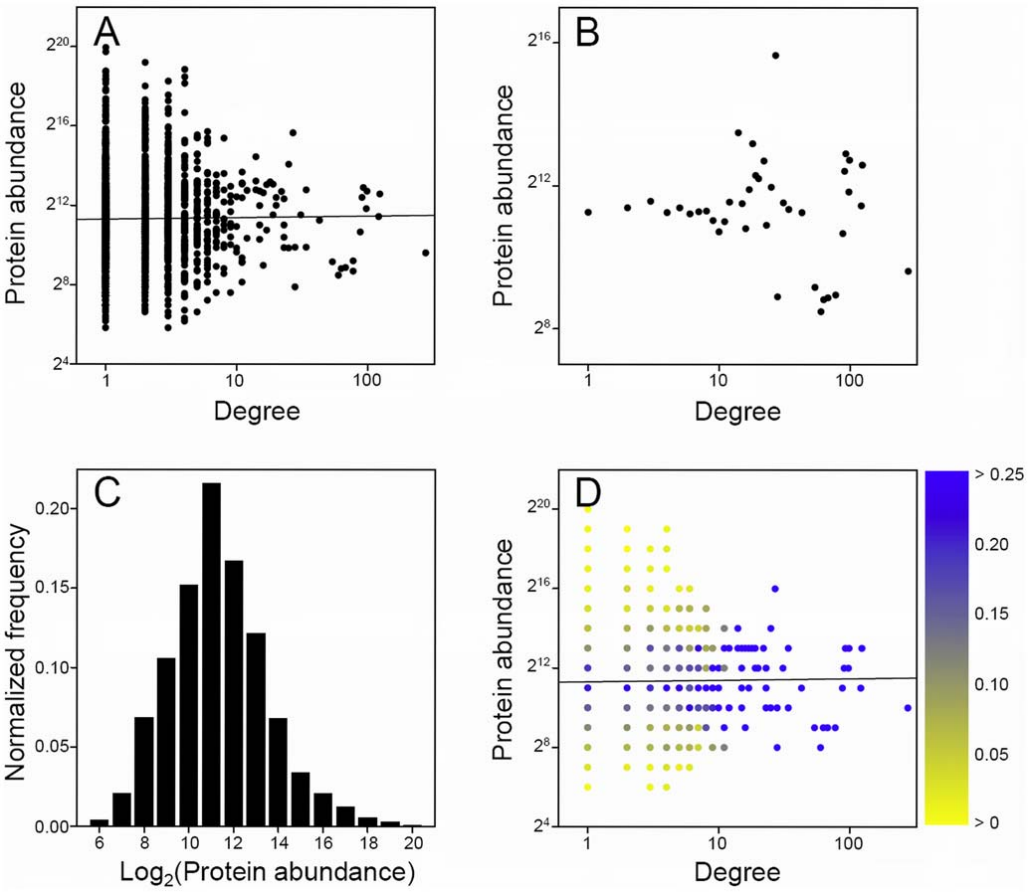
Relationship between protein degree and abundance in the raw yeast Y2H Ito PPI network [7] using the western blot abundance measurements of Ghaemmaghami et al. [42]. (A)–(D), see Figure 1 legend.

To gauge the possibility of artificial correlations arising from irregular abundance distributions of proteins in the PPI data sets, we computed abundance distributions for proteins grouped by degree. These are illustrated via color maps (Figures 1, 2, 3).

Correlations between essentiality and degree were evaluated by calculating *P*-values for two-sample Kolmogorov-Smirnov (KS) test for differences between degree distributions of essential and nonessential proteins. Comparable *P*-values were determined to test for correlations between protein abundance and essentiality.

The enrichment of essential-essential protein interactions in a network was evaluated by computing a *P*-value, via calculation of a *Z*-score and assuming a normal distribution, for the difference between the actual number and the average obtained from randomly selecting proteins to be essential. However, when randomly selecting proteins to be considered essential, we ensured that the total number and the degree distribution of the chosen essential proteins matched those of the actual essential proteins. This ensures that artificial differences arising from changes in degree distributions of essential proteins are not observed whilst simultaneously conserving the network structure. Results were deduced from 1000 simulations.

## Results

### Influence of Protein Abundance upon Degree in Raw PPI Data Sets

Experimental PPI data sets are derived from techniques that attempt to detect the presence of protein associations in a cellular environment. Therefore, an investigation into any influences the cellular concentration of each protein has on the detected interactions should be based on protein abundance measurements. Recent determinations of yeast cellular protein abundances during normal growth were achieved by tagging open reading frames (ORFs), thereby allowing expressions from their natural chromosomal locations, and measuring absolute levels by WB [42] and FC [43] techniques. We utilized protein abundance measurement data sets from both of these studies. Although it is the proteins that are overwhelmingly responsible for the various cellular functions, expression levels of their precursors, mRNA molecules, provide considerable insights into the internal states of a cell. In fact, measurement of mRNA expression levels by microarray experiments [51]–[53] is far simpler and more widespread than measurement of protein abundances. Accordingly, there have been an enormous number of GE studies that have determined yeast mRNA abundances during normal growth. The findings reported here utilized the GE measurements of Holstege et al. [44]; however, it should be stressed that comparable results were obtained when using GE measurements from three more-recent studies [54]–[56]. The previously noted trend between degree and abundance [35] was based on the data of Holstege et al. [44]. As noted above, six raw yeast PPI data sets were investigated here encompassing three diverse HT technologies: TAP (three data sets), HMS-PCI (one data set), and Y2H (two data sets). We have also investigated two raw TAP data sets for *E. coli* and used GE measurements from three studies [48]–[50].

Table 1 shows the correlations between yeast protein degree and abundance, for each of the WB, FC, and GE measurements. We find that all three raw TAP PPI data sets contain statistically significant correlations between protein degree and abundance. The Pearson correlation coefficients for test of linear relationship between log values, lying between 0.23 and 0.33, are similar but not large and all corresponding Spearman rank correlation coefficients are very close in value. However, all *P*-values are less than 0.0001, suggesting that the relationships are significant, i.e., they do not represent random events. We stress that these correlations are obtained without any averaging of data, i.e., each protein's degree and abundance is included as a single data entity. Merging the Gavin and Krogan-TOF data sets produces a PPI network that contains very similar degree/abundance correlations to the individual data sets. Figure 1A, showing all data points, illustrates the degree/abundance relationship in the raw TAP Gavin PPI network using the WB abundance measurements [42], and Figure 1B shows the general trend where log_2_(abundance) values were averaged over proteins having the same degree. The Pearson correlation coefficient for this latter averaged data is 0.52 and the corresponding *P*-value is less than 0.0001. Similar plots are obtained for the other raw yeast TAP PPI data sets and also when using the FC (protein) and GE (mRNA) abundance measurements. The results for the *E. coli* TAP data sets are almost identical to those of the yeast TAP sets (Table 2), with Pearson and Spearman correlation coefficients lying between 0.11 and 0.46 and all associated *P*-values less than 0.0001. Figures 2A and 2B show the non-averaged and averaged data, respectively, for the Butland PPI network using the GE measurements of Covert et al. It is clear that there is a definite propensity, although slight, for proteins of increasing degree to have higher abundances.

**Table 1:** Pearson correlation coefficients (*r*) and corresponding *P*-values for tests of linear association between log(degree) and log_2_(abundance) in raw yeast PPI data sets.

PPI data set	Western blot (Ghaemmaghami et al.)			Flow cytometry (Newman et al.)			Gene expression (Holstege et al.)		
	N_P_ a	*r*	*P* b	N_P_ a	*r*	*P* b	N_P_ a	*r*	*P* b
*TAP*									
Gavin	2146	0.29 (0.28)	<	1585	0.29 (0.26)	<	2496	0.33 (0.30)	<
Krogan-TOF	2291	0.24 (0.21)	<	1523	0.25 (0.20)	<	2701	0.23 (0.21)	<
Krogan-LCMS	3543	0.26 (0.21)	<	2314	0.28 (0.22)	<	4707	0.26 (0.20)	<
Gavin+Krogan-TOF^c^	2845	0.33 (0.31)	<	1925	0.32 (0.27)	<	3410	0.32 (0.30)	<
*HMS-PCI*									
Ho	1326	0.26 (0.23)	<	938	0.28 (0.25)	<	1630	0.18 (0.17)	<
*Y2H*									
Ito	2107	0.01 (0.04)	0.5820	1357	−0.05 (−0.04)	0.0646	2910	∼0 (0.01)	0.9212
Uetz	929	−0.01 (0.01)	0.8470	599	−0.07 (−0.06)	0.0686	1221	−0.04 (−0.03)	0.2214
Ito+Uetz^d^	2403	−0.01 (0.01)	0.6066	1560	−0.06 (−0.04)	0.0277	3311	−0.01 (−0.01)	0.5689

Spearman rank correlation coefficients are provided in parentheses.

a Number of proteins in PPI data set having abundance measurements.

b The symbol “<” signifies *P*<0.0001 for both Pearson and Spearman correlation coefficients.

c Combined Gavin and Krogan-TOF interaction data sets.

d Combined Ito and Uetz interaction data sets.

**Table 2:** Pearson and Spearman rank (in parentheses) correlation coefficients (*r*) for tests of linear association between log(degree) and log_2_(mRNA expression) in raw *E. coli* TAP PPI data sets.

PPI data set	Covert et al.		Kang et al.		Salmon et al.	
	N_P_ a	*r*	N_P_ a	*r*	N_P_ a	*r*
Butland	1277	0.46 (0.45)	1278	0.45 (0.42)	856	0.24 (0.21)
Arifuzzaman	2918	0.21 (0.19)	2919	0.21 (0.20)	1764	0.13 (0.11)
Butland+Arifuzzman^b^	3158	0.30 (0.28)	3159	0.30 (0.27)	1904	0.17 (0.13)

All *P*<0.0001 for both Pearson and Spearman correlation coefficients.

a Number of proteins in PPI data set having abundance measurements.

b Combined Butland and Arifuzzaman interaction data sets.

The observed correlations could be artificial if the abundance distributions of the proteins at each degree are skewed. However, this notion can be discounted as we are finding statistically significant correlations for non-averaged data. Nonetheless, we investigate the abundance distributions by degree to further establish the authenticity of the degree/abundance relationship in the raw TAP PPI networks. For proteins appearing in both the raw Gavin PPI and WB abundance measurement data sets, we show the total abundance distribution (binned in integer values of log_2_(abundance)) in Figure 1C and for each degree (as a color map) in Figure 1D. The total abundance distribution is seen to be very close to normal and the distributions for each degree are also reasonably symmetric about their averages. Note that Figure 1D reflects the general trend of the averaged data in Figure 1B. These observations are echoed for the *E. coli* TAP Butland PPI network and the GE measurement data set of Covert et al. (Figure 2C and D), where the degree/abundance relationship appears more pronounced. Therefore, it is possible that the TAP method is detecting interactions that are influenced by the cellular concentrations of the proteins. While this finding may not be surprising, as the TAP method expresses tagged bait and potential prey ORFs from their natural chromosomal locations, it does imply that raw TAP-determined PPI networks incorporate a probabilistic, or random, element. The higher the cellular abundance of a protein, the more often it is likely to be detected in purifications and, therefore, the more interactions it will be construed to be involved in. Although the correlation coefficients given here are not large, it is known that mRNA and protein abundance measurements contain many sources of variation due to technical and biological factors.

The HMS-PCI technique to isolate and identify co-purifying proteins [6] is very similar to that used in the TAP studies [5], [8], as it uses an affinity purification method to isolate complexes. However, rather than express tagged ORFs from their native environments, as in the TAP studies, Ho et al. express tagged ORFs from plasmids containing *GAL1* promoters. It is unclear how this latter non-native delivery system affects the cellular abundances of tagged baits but one might expect the prey proteins to have concentrations similar to those of normal growth conditions. Not surprisingly, perhaps, we also find statistically significant correlations between degree and abundance for the Ho PPI data set. Correlation coefficients are again modest, lying between 0.17 and 0.28 (Table 1), but all *P*-values are less than 0.0001, suggesting a statistically significant relationship between degree and abundance for the Ho data set. Therefore, it would seem that general affinity purification methods are detecting interactions that are somewhat mediated by the proteins' cellular abundances.

Without counter-example PPI data sets that show no correlation between degree and abundance, one might expect the findings here to be biologically relevant, i.e., on the average, the number of interactions a protein is involved in is related to its cellular concentration. Such an interpretation would suggest that a salient probabilistic element exists in the interactome. However, regardless of the extent of the probabilistic behavior, it is well established that there exist stable protein complexes [47]. Therefore, it is of interest that we find all of the raw Y2H PPI data sets, whether individual Ito, Uetz, or combined, to have no correlation between degree and abundance (Table 1). All correlations coefficients are very small, lying between −0.07 and 0.04, and all *P*-values, for Pearson correlation coefficients, are greater than 0.02, although for the individual Ito and Uetz data sets they range from 0.06 to 0.92. When using the GE and WB abundance measurements, correlation coefficients have absolute values of less than 0.05 and *P*-values are greater than 0.22. *P*-values are smallest for the FC abundance measurements and the reasons why are not immediately clear. Figures 3A and 3B show the non-averaged and averaged data, respectively, for the Ito PPI network using the WB protein abundance measurements. It is clear from both plots that no relationship exists between degree and abundance.

In order to further clarify the differences between PPI networks determined from the Y2H and the affinity purification methods we analyzed abundance distributions by degree for proteins appearing in both the raw Ito PPI and WB abundance measurement data sets. Figures 3C and 3D show the total abundance distribution and the distributions for each degree, respectively. The total abundance distribution is essentially normal and notably very similar to that of the TAP Gavin PPI data set (Figure 1C). Additionally, Figure 3D shows that the distributions for each degree are symmetric about their averages and also clearly illustrates the lack of a degree/abundance correlation. Therefore, when compared against the results for the raw TAP and HMS-PCI data sets, we must conclude that the raw Y2H PPI data sets contain no degree/abundance relationship. Furthermore, we must also affirm that the degree/abundance correlations observed in the TAP and HMS-PCI PPI networks are not the result of skewed abundance distributions, whether total or for individual degrees. It is clear that the Y2H methodology is distinct from the affinity purification methods in that protein expression levels do not influence the observed interactions for the former technique.

The findings above are consistent considering that in a Y2H screen both bait and prey proteins are expressed from similar plasmids. A protein expressed from a plasmid is likely to have a different cellular concentration than if it were expressed from its native chromosomal location. Additionally, different proteins expressed from the same plasmid are presumed to have similar expression levels. However, if the latter were not true, then it is possible that the Y2H screen could be influenced by some other abundance factor related to post-transcriptional modification. Investigation of this speculation is not straightforward but may be possible if the translational efficiencies of the proteins can be estimated.

### Influence of Protein Abundance upon Degree in HC PPI Data Sets

Some of the yeast PPI studies utilized in this work also inferred HC interaction data sets from their raw data. These HC PPI data sets are meant to contain interactions that are most reproducible or resolute. The methods used to infer HC interactions were varied (see Materials and Methods). Therefore, it is of interest to discover whether these sets contain any relationship between degree and abundance. We find that all the yeast HC PPI networks deduced from raw affinity purification data (TAP and HMS-PCI) contain statistically significant correlations (Table 3). All correlation coefficients are modest, lying between 0.13 and 0.39, and are similar to those observed for the raw TAP and HMS-PCI data sets. However, all *P*-values are less than 0.0001, again suggesting nontrivial relationships exist between degree and abundance. These results are surprising for the Krogan-CORE, Krogan-INT, and HC Ho data sets, as their inferring methodologies included, as a first step, the removal of promiscuous proteins. Krogan et al. removed 44 nonspecific contaminants and nearly all cytoplasmic ribosomal subunits from the raw data as a preliminary step, while the HC Ho PPI data set is a subset of the raw data in which nonspecifically binding proteins have been subtracted. In spite of these removals, and any further inferring procedures, their HC data sets still contain a degree/abundance relationship, albeit weak. Therefore, the influence of protein cellular abundance is not limited to a small proportion of highly-abundant and promiscuous proteins. Rather, the effect seems subtly ingrained throughout the data. The TAP HC data set of Gavin et al. was inferred by first determining ‘socio-affinity’ scores for each pair of proteins, which quantified the propensity of them to occur together in purifications. However, degree/abundance correlation coefficients lie between 0.31 and 0.39. In fact, the HC Gavin data set has larger correlation coefficients than the corresponding values for the raw PPI network. These findings for the HC TAP and HMS-PCI data sets might suggest that the degree/abundance relationship is, as discussed earlier, biologically significant. Alternatively, the observed correlations for the HC data sets could be the result of inferring procedures that are not completely effectual.

**Table 3:** Pearson correlation coefficients (*r*) and corresponding *P*-values for tests of linear association between log(degree) and log_2_(abundance) in high-confidence yeast PPI data sets.

PPI data set	Western blot (Ghaemmaghami et al.)			Flow cytometry (Newman et al.)			Gene expression (Holstege et al.)		
	N_P_ a	*r*	*P* b	N_P_ a	*r*	*P* b	N_P_ a	*r*	*P* b
Gavin *[TAP]*	1286	0.31 (0.30)	<	1012	0.38 (0.37)	<	1470	0.39 (0.37)	<
Krogan-CORE *[TAP]*	2208	0.20 (0.18)	<	1461	0.16 (0.14)	<	2578	0.22 (0.21)	<
Krogan-INT *[TAP]*	1081	0.15 (0.13)	<	778	0.16 (0.15)	<	1186	0.20 (0.19)	<
Ho *[HMS-PCI]*	1240	0.26 (0.26)	<	874	0.26 (0.26)	<	1535	0.21 (0.21)	<
Ito *[Y2H]*	596	−0.02 (−0.01)	0.6514	380	−0.06 (−0.06)	0.2393	727	0.02 (0.03)	0.6726

Spearman rank correlation coefficients are provided in parentheses.

a Number of proteins in PPI data set having abundance measurements.

b The symbol “<” signifies *P*<0.0001 for both Pearson and Spearman correlation coefficients.

The Y2H HC data set of Ito et al. is a subset of the raw PPI network and includes only those interactions that were experimentally detected at least three times. We find that this interaction set shows no correlation between degree and abundance (Table 3). This is not surprising as the raw data set also contains no relationship. The correlation coefficients are small, ranging from −0.06 to 0.03, and all *P*-values for Pearson correlation coefficients are larger than 0.23.

The results for the HC PPI data sets are identical to those of the raw data sets. Those derived from affinity purification experiments show weak, but statistically significant correlations between degree and abundance, while all Y2H PPI data sets show no relationship between degree and abundance. These findings further exemplify the difference between interactions detected by Y2H screens and affinity purification procedures.

### Relation between Essentiality and Topology in Raw PPI Data Sets

The correlation between degree and essentiality, in that proteins having more interactions are more likely to be essential, has been noted previously [20]–[23] and is generally an accepted precept known as the centrality-lethality rule. Recent analysis of curated, inferred, and HC yeast networks show that essential proteins are more likely to be involved in essential complex biological modules and, therefore, their degrees are on the average higher [23]. However, the findings presented here, that PPI networks determined using affinity purification procedures (TAP and HMS-PCI) have statistically significant correlations between degree and abundance while Y2H PPI networks do not, warrants another look at the degree/essentiality property.

**Figure 4:**
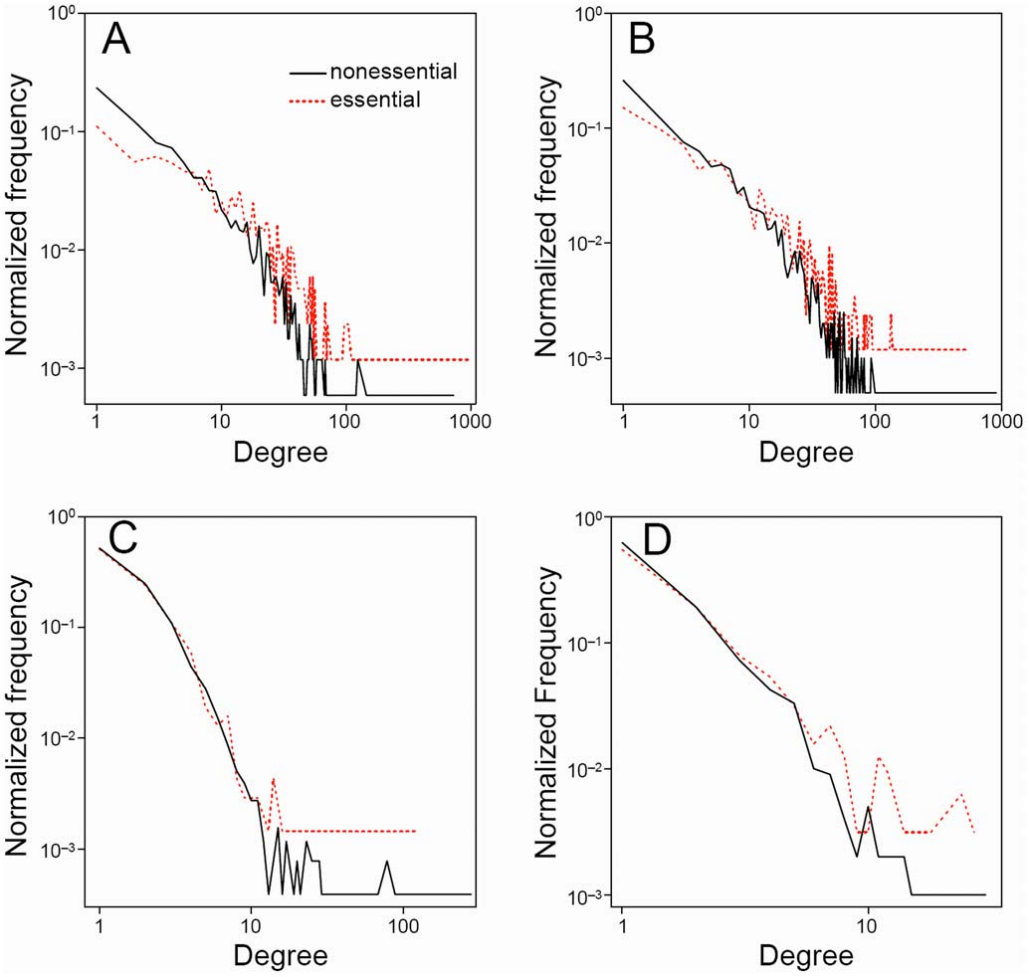
Degree distributions of essential (red dashed) and nonessential (black) proteins in raw yeast PPI networks. (A) Gavin (TAP) [5], (B) Krogan-TOF (TAP) [8], (C) Ito (Y2H) [7], (D) Uetz (Y2H) [9].

Table 4 gives average degrees of essential and nonessential proteins in the raw yeast PPI networks. We find that essential proteins have higher average degrees than nonessential proteins in all of the raw PPI networks. However, it is known that degree distributions of PPI networks are not normal; rather, they resemble power-law scaling [22], [57]. Therefore, in order to determine the significance of the difference between degrees of essential and nonessential proteins we use the two-sample KS test to compare their degree distributions. We find that degree distributions of essential proteins in the TAP and HMS-PCI data sets are significantly different to those of nonessential proteins, with all *P_KS_*-values being less than 0.0001 (Table 4). These differences are illustrated in Figures 4A and 4B, which show degree distributions of essential and nonessential proteins in the raw Gavin and Krogan-TOF PPI data sets, respectively.

**Table 4:** Tests of difference beween degree distributions of essential and nonessential proteins in raw and high-confidence yeast PPI data sets.

PPI data set	Essential^a^	Nonessential^b^	*P_KS_* c	Correlation between degree/abundance
*Raw*				
Gavin *[TAP]*	20.79	11.02	<	yes
Krogan-TOF *[TAP]*	18.02	10.63	<	yes
Krogan-LCMS *[TAP]*	28.49	14.36	<	yes
Ho *[HMS-PCI]*	12.24	8.18	<	yes
Ito *[Y2H]*	3.04	2.60	0.9915	no
Uetz *[Y2H]*	2.59	1.96	0.1542	no
*High-confidence*				
Gavin *[TAP]*	5.88	5.90	0.5160	yes
Krogan-CORE *[TAP]*	7.82	4.20	<	yes
Krogan-INT *[TAP]*	5.21	2.95	<	yes
Ho *[HMS-PCI]*	6.04	3.89	<	yes
Ito *[Y2H]*	2.13	1.84	0.9994	no

a Average degree of essential proteins.

b Average degree of nonessential proteins.

c *P*-value for two-sample Kolmogorov-Smirnov test for difference between degree distributions of essential and nonessential proteins. The symbol “<” signifies *P*<0.0001.

In stark contrast, we find that degree distributions of essential and nonessential proteins in the raw Y2H networks are not significantly different, with *P_KS_*-values of 0.9915 and 0.1542 for the raw Ito and Uetz data sets, respectively. Degree distributions of essential and nonessential proteins in the Ito and Uetz PPI networks are shown in Figures 4C and 4D, respectively, and it is clear that for both data sets the curves are very similar. Therefore, we conclude that the raw Y2H data sets show no correlation between degree and essentiality. In fact, the raw Ito data set has a *P*-value very near to one. It has been previously reported that the Ito data set has a weak correlation between degree and essentiality [20]; however, we find no difference between degree distributions of essential and nonessential proteins for this data set.

The degree/essentiality relationships discussed above for the raw PPI data sets are curious in that if there is a (weak) degree/abundance correlation, there is also a degree/essentiality relationship. These tandem correlations are observed in all of the raw interaction data sets determined by affinity purification methods (TAP and HMS-PCI). The converse is also true, in that, if there is no degree/abundance relationship, there is also no degree/essentiality correlation (Table 4). These tandem non-correlations are observed in the Y2H interaction data sets. Insights into why these correlations are associated can be obtained by looking for a relationship between essentiality and abundance. In fact, we find that essential proteins are more abundant than nonessential proteins in all of the yeast abundance measurements utilized here. *P*-values, assuming normal distributions, for tests of difference between average log_2_(abundance) of essential and nonessential proteins for the WB [42], FC [43], and GE [44] measurements are 10^−19^, 10^−7^, and 10^−37^ respectively. *P*-values for two-sample KS tests are very similar, 10^−18^, 10^−8^, and 10^−31^ for WB, FC, and GE measurements, respectively. Therefore, the correlation between degree and essentiality in the raw TAP and HMS-PCI networks may be artificial due to essential proteins generally being more abundant. This would explain why there is no correlation between degree and essentiality in the Y2H data sets as they also contain no correlations between degree and abundance. Therefore, the common notion that essential proteins generally have higher degrees than nonessential proteins may be misleading.

In an effort to identify deterministic, or nonrandom, signatures in the raw yeast PPI networks, we quantified the enrichment of essential-essential interactions in the data sets by comparing the observed numbers with those from strict randomized simulations. For a given PPI network, proteins to be considered as essential were selected at random with the constraint that the degree distribution of the selected proteins matched those of the actual essential proteins. This ensures that the results are not perturbed by varying degree distributions of the ‘essential’ proteins whilst simultaneously conserving the network structures. Data were deduced from 1000 realizations and the results are given in Table 5. We consistently find that the numbers of actual essential-essential interactions are larger than those from the randomized simulations and that standard deviations are relatively small. Accordingly, all *P*-values, being less than 0.0001, indicated significant nonrandom enrichments. Therefore, biological signatures seem evident in all raw yeast PPI data sets, including Y2H despite that these networks show no correlation between degree and essentiality.

**Table 5:** Enrichment of interactions between essential proteins in raw and high-confidence yeast PPI data sets.

PPI data set	Actual	Random^a^	*σ* b
*Raw*			
Gavin *[TAP]*	4692	4310	32.6
Krogan-TOF *[TAP]*	3416	3203	30.9
Krogan-LCMS *[TAP]*	6566	6235	52.7
Ho *[HMS-PCI]*	1470	1391	20.2
Ito *[Y2H]*	315	248	12.4
Uetz *[Y2H]*	163	119	7.8
*High-confidence*			
Gavin *[TAP]*	892	829	14.1
Krogan-CORE *[TAP]*	1742	1397	24.0
Krogan-INT *[TAP]*	906	765	13.4
Ho *[HMS-PCI]*	716	652	14.9
Ito *[Y2H]*	97	68	6.2

All *P*<0.0001.

a Average number of interactions observed between essential proteins for 1000 simulations in which essential proteins were selected randomly from all proteins occurring in a network. In each simulation, the total number of selected proteins and their degree distribution were constrained to be identical to those of the actual essential proteins.

b Standard deviation for simulations described above.

### Relation between Essentiality and Topology in HC Data Sets

Table 4 also shows the average degrees of essential and nonessential proteins in the HC yeast PPI networks together with *P*-values from two-sample KS tests. The Y2H HC Ito data set, with a *P*-value of essentially one, has no correlation between degree and essentiality. This result is almost identical to that of the raw Ito PPI network, indicating that the Y2H method does not bias essential proteins to have more interacting partners. The HC Krogan data sets, like their raw counterparts, show significant correlations (all *P*-values are less than 0.0001) between degree and essentiality. This result is unsurprising as these data sets also show a relationship between degree and abundance. Similar findings are obtained for the HC Ho data set (HMS-PCI). Up to this point, the findings for HC data sets lend support to the notion that any identified correlation between degree and essentiality in a PPI network may be artificially induced, as essential proteins are generally more abundant than nonessential. In stark contrast, however, the HC Gavin data set, with a *P*-value of 0.52, shows no correlation between degree and essentiality although it does contain a degree/abundance association. Of the PPI networks investigated in this work, the HC Gavin data set is the only one that contains a degree/abundance correlation but not a degree/essentiality relationship. The reasons for this are not immediately clear but are presumably related to the steps in the HC interaction inferring procedure.

We find that all of the HC yeast PPI networks show enriched interactions between essential proteins (Table 5). All *P*-values are less than 0.0001, indicating that the observed numbers of essential-essential interactions are significantly larger than from the strict randomized simulations. Our test for enrichment is very strict in that we freeze the network structure and degree distributions of essential (actual and randomly chosen) proteins and, therefore, it is difficult to form any extensive topological insights. However, our tests indicate, without question, that all raw and HC yeast PPI networks show a propensity for essential proteins to prefer to interact with each other. We deduce that a biological signature in a PPI network does not appear in the commonly acknowledged form of a degree/essentiality correlation; rather it manifests itself by enhancing interactions between essential proteins. While a recent study concludes that in HC PPI networks essential proteins are more likely to be involved in essential complex biological modules [23], here we find the more general case that essential proteins prefer to interact with each other.

## Discussion

It is shown that raw and HC TAP and HMS-PCI PPI networks contain statistically significant correlations between protein degree and abundance. The previously noted trend between protein degree and mRNA abundance [35] is confirmed here using protein abundances and more extensive analyses. The results are consistent for yeast (three TAP and one HMS-PCI) and *E. coli* (two TAP) data sets. For yeast, the correlations are similar for three diverse protein and mRNA abundance measurement technologies: western blot, flow cytometry, and gene expression. For *E. coli*, the results are consistent when using gene expression measurements during normal aerobic growth from three studies. Although correlation coefficients are modest, the observations are highly significant. Furthermore, protein abundance and gene expression measurements are known to be variable. Yet, the identified correlations between degree and abundance are consistently observed and indicate an inherent and nontrivial property of the data.

The TAP method extracts tagged bait proteins, expressed from their native genome locations, and determines which other proteins, or preys, have co-purified, or complexed, with them. The HMS-PCI method is similar except that tagged bait proteins are expressed from plasmids. In both techniques the prey proteins are expressed under natural conditions and from their native environments. As such, the degree/abundance relationship in TAP and HMS-PCI PPI data sets may not be wholly unexpected. If all protein pairs have very similar binding affinities, then probability theory dictates that the number of detected interactions for the proteins will correlate roughly with their concentrations, or abundances. Nonrandom influences in the forms of differing expression times and cellular locations will remove some of the probabilistic elements. While it is known that the TAP method induces retrieval of nonspecific contaminants, or promiscuous prey proteins, we find that their promiscuity may be an artificial property induced by their high abundances. Statistically significant correlations between degree and abundance are also observed in inferred HC TAP and HMS-PCI data sets. While some of the inferring procedures involved steps to eliminate contaminant and nonspecifically binding proteins, the resulting HC interaction data sets still contain degree/abundance relationships. Therefore, the influence of protein cellular abundance is subtly ingrained throughout the data and not limited to a small proportion of highly abundant and promiscuous proteins.

In direct contrast to the TAP and HMS-PCI data sets, the raw and HC yeast Y2H PPI networks show no correlation between degree and abundance. These results identify a systematic difference between PPI networks determined from the Y2H and affinity purification platforms. In hindsight, this is consistent with the experimental design. The Y2H approach expresses a pair of bait and prey proteins, to be tested for an interaction, from engineered plasmids. Therefore, their expression levels are likely to be different than in their natural environments. That is not to say that the Y2H method is not influenced by protein abundance in some way. It is generally accepted that proteins expressed from the same plasmid have similar abundances. While their expressions may be similar, their translational efficiencies may not be and, if so, it is possible that Y2H screens are affected by plasmid-induced abundances. However, investigation of this premise is not straightforward. Nonetheless, we find here that Y2H PPI data sets are not in any way mediated by protein cellular abundance.

The lack of degree/abundance correlations in Y2H PPI data sets can be related to the findings of Zhang et al. [58], who show that interactions in the Y2H data of Ito et al. [7] are more likely to be biologically functional (i.e., independently reported in two or more publications using non-Y2H techniques) if the participating proteins have relatively high *in vivo* abundances [58]. Taken together, the results imply that while proteins having high abundances may be detected by the Y2H approach to have few interactions, those interactions are more likely to be specific. Conversely, while proteins having low abundances may have many Y2H-detected interactions, they are more likely to be non-specific. Since *in vivo* abundances of many proteins are often less than when tagged in Y2H experiments, the detected interactions may not necessarily occur in the natural environment. However, associations detected between proteins having high *in vivo* abundances, while not guaranteed to be relatively many, are more likely to be specific and naturally occurring. Therefore, while *in vivo* abundances do not influence the total number of Y2H-detected interactions for each protein, they intrinsically impact the numbers that are specific.

In light of the observed (weak) correlations between degree and abundance for the TAP and HMS-PCI PPI networks, we reinvestigated the centrality-lethality rule, which implies that proteins having more interactions are more likely to be essential. From analysis of three diverse yeast protein and mRNA abundance measurement data sets we find that essential proteins are more prevalent than nonessential proteins. We also observed that generally degree/abundance relationships occur in tandem with degree/essentiality correlations. Additionally, whenever there is no degree/abundance association, there is also no degree/essentiality correlation. Therefore, the degree/essentiality correlations in the TAP and HMS-PCI data sets seem artificial. The lack of any degree/essentiality correlation in the Y2H data sets supports this notion. The results imply caution in accepting the generally acknowledged centrality-lethality rule.

Biological, or nonrandom, signatures were identified in all of the PPI networks in the form of enrichments of interactions between essential proteins. This propensity for essential proteins to interact with each other was deduced by comparisons with strict randomized simulations. Therefore, we deduce that essentiality does not manifest itself as a biological property in the commonly acknowledged form of a degree/essentiality correlation; rather, it is actualized by the enhancement of interactions between essential proteins.

As well as demonstrating systematic differences in PPI networks determined using the Y2H and affinity purification methodologies, we discern the nature of the probabilistic element in the latter approaches. These findings should provide insights into the design of more effective strategies to deduce the specific and invariable interactions from raw TAP and HMS-PCI data sets. Such unbiased, or untrained, procedures are vital if we are to infer HC PPI networks for organisms other than yeast and exploit them to discern genuine biological traits and features. One avenue of discovery that is receiving recent attention is the development of analyses that combine gene expression and PPI data sets. For given conditions, whether environmental or physiological, changes in mRNA levels relative to a reference state are mapped onto the PPI networks in order to identify response-type modules or sub-networks. In this respect, it is vital to comprehend the underlying nature of the PPI data set. Highly abundant proteins are likely to have larger fluctuations in their expression levels and, therefore, if one is utilizing a network deduced from an affinity purification procedure, care must be taken when interpreting the results.
